# Clinical mechanism of muscle mass loss during neoadjuvant chemotherapy in older patients with esophageal cancer: a prospective cohort study

**DOI:** 10.1093/dote/doae096

**Published:** 2024-11-05

**Authors:** Tsuyoshi Harada, Tetsuya Tsuji, Junya Ueno, Nobuko Konishi, Takumi Yanagisawa, Nanako HIjikata, Aiko Ishikawa, Kakeru Hashimoto, Hitoshi Kagaya, Noriatsu Tatematsu, Sadamoto Zenda, Daisuke Kotani, Takashi Kojima, Takeo Fujita

**Affiliations:** Department of Rehabilitation Medicine, National Cancer Center Hospital East, Kashiwa, Chiba, Japan; Department of Rehabilitation Medicine, Keio University Graduate School, Shinjuku, Tokyo, Japan; Department of Rehabilitation Medicine, National Cancer Center Hospital East, Kashiwa, Chiba, Japan; Department of Rehabilitation Medicine, Keio University School of Medicine, Shinjuku, Tokyo, Japan; Department of Rehabilitation Medicine, National Cancer Center Hospital East, Kashiwa, Chiba, Japan; Department of Rehabilitation Medicine, National Cancer Center Hospital East, Kashiwa, Chiba, Japan; Department of Rehabilitation Medicine, National Cancer Center Hospital East, Kashiwa, Chiba, Japan; Department of Rehabilitation Medicine, National Cancer Center Hospital East, Kashiwa, Chiba, Japan; Department of Rehabilitation Medicine, National Cancer Center Hospital East, Kashiwa, Chiba, Japan; Department of Rehabilitation Medicine, Keio University School of Medicine, Shinjuku, Tokyo, Japan; Department of Rehabilitation, National Center for Geriatrics and Gerontology, Obu, Aichi, Japan; Department of Rehabilitation, National Center for Geriatrics and Gerontology, Obu, Aichi, Japan; Department of Integrated Health Sciences, Nagoya University, Nagoya, Aichi, Japan; Department of Radiation Oncology, National Cancer Center Hospital East, Kashiwa, Chiba, Japan; Department of Gastrointestinal Oncology, National Cancer Center Hospital East, Kashiwa, Chiba, Japan; Department of Gastrointestinal Oncology, National Cancer Center Hospital East, Kashiwa, Chiba, Japan; Department of Esophageal Surgery, National Cancer Center Hospital East, Kashiwa, Chiba, Japan

**Keywords:** chemotherapy, esophageal cancer, geriatrics, nutrition, rehabilitation

## Abstract

In older patients with locally advanced esophageal cancer (LAEC), loss of skeletal muscle mass during neoadjuvant chemotherapy (NAC) is associated with poor clinical outcomes. This study aimed to investigate factors associated with loss of skeletal muscle mass during NAC in older patients with LAEC. This was a single-center exploratory prospective cohort study. Consecutive patients aged ≥65 years with LAEC scheduled for curative esophagectomy after NAC were enrolled between October 2021 and December 2023. As a primary endpoint, loss of skeletal muscle mass index (ΔSMI: pre-NAC minus post-NAC value) was calculated from computed tomography images before and after NAC. Significant pre-NAC and during-NAC factors with ΔSMI were detected with a multivariate regression model. Statistical significance was considered as two-tailed *P* <0.05. A total of 69 patients were analyzed. The mean age was 72.9 years, and 53 (77%) were male. Mean SMI before and after NAC was 43.1 and 40.9 cm^2^/m^2^, and mean ΔSMI was 2.2 cm^2^/m^2^. In multivariate analysis, ΔSMI was associated with increased sitting time during NAC (per 1 min/day, adjusted coefficient 0.007, 95% confidence interval [CI] 0.001 to 0.013, *P* = 0.016), decreased Geriatric Nutritional Risk Index during NAC (per 1 score, adjusted coefficient −0.146, 95% CI −0.213 to −0.013, *P* = 0.002), and worsening decreased appetite during NAC (vs. no worsening, adjusted coefficient 1.571, 95% CI 0.279 to 2.862, *P* = 0.018). It was hypothesized that the inactivity-related mechanism and malnutrition-related mechanism are important for skeletal muscle mass loss during NAC in older patients with LAEC.

## INTRODUCTION

Death caused by gastrointestinal cancer was reported to account for more than one-third (35%) of all cancer-related deaths in 2018.^1^ In particular, locally advanced esophageal cancer (LAEC) has a poor prognosis, with a reported 5-year survival rate of <70%.^2^ One of the global standard treatments for patients with LAEC is esophagectomy following neoadjuvant chemotherapy (NAC).^2^^,^^3^ However, esophagectomy following NAC has negative impacts on physical function, skeletal muscle mass, and quality of life^4–6^ because this multidisciplinary cancer treatment is one of the most intense and demanding treatments available. The number of patients aged ≥65 years with LAEC has recently increased globally.^7^ During cancer treatment, these older patients carry several risks for complications, adverse events, and dependence in daily life.^8^^,^^9^ Therefore, there is an international need for evidential information to develop novel supportive care to prevent complications and adverse events of esophagectomy following NAC and to improve clinical outcomes, including health-related quality of life, in older patients with LAEC.^10^

Skeletal muscle mass has an impact on physical function, disability, and quality of life, important factors in older patients with cancer.^11^^,^^12^ It was reported that the loss of skeletal muscle mass during NAC not only causes long-term delayed recovery of skeletal muscle mass but also negatively impacts postoperative pneumonia and 3-year survival rate through preoperative progression of physical frailty in older patients with LAEC.^4^^,^^13^ However, there is not only no positive clinical trial of effect of prehabilitation during NAC to prevent the loss of skeletal muscle mass but also no research on the clinical mechanism of the loss of skeletal muscle mass during NAC in older patients with LAEC. Such information regarding clinical mechanisms contributes to the development of novel supportive care to prevent skeletal muscle mass loss and the progression of physical frailty during chemotherapy in older patients with cancer. This prospective cohort study aimed to investigate factors associated with loss of skeletal muscle mass during NAC in older patients with LAEC and thereby determine the clinical mechanism.

## METHODS

### Design and participants

This was a single-center exploratory prospective cohort study in a Japanese national cancer center. A total of 80 patients aged ≥65 years with clinical stage IB, II, III, or IV esophageal cancer without distant organ metastasis (Union for International Cancer Control tumor–node–metastasis [UICC–TNM] classification, 8th edition), who were scheduled for curative esophagectomy after NAC at the National Cancer Center East Hospital in Japan between October 2021 and December 2023 without untreated or undertreated duplicate cancer upon starting NAC, were consecutively included. Exclusion criteria were alteration of the treatment such as changing to chemoradiotherapy because of progression tumor during NAC, using a feeding tube in nutritional therapy, dropout of NAC, and clinical decline. This study was approved by the Research Ethics Committee of the National Cancer Center (2021-179) in accordance with the Declaration of Helsinki. All participants received verbal and written explanations regarding study procedures and signed an informed consent form upon agreement to participate.

### NAC and surgery

The NAC regimen was cisplatin + fluorouracil (FP), oxaliplatin + leucovorin + fluorouracil (FOLFOX), or docetaxel + cisplatin + fluorouracil (DCF). The FP regimen consisted of cisplatin (80 mg/m^2^) on day 1 and fluorouracil (800 mg/m^2^) on days 1–5 of a 21-day cycle. The FOLFOX regimen consisted of oxaliplatin (85 mg/m^2^), leucovorin (200 mg/m^2^), and a bolus of fluorouracil (400 mg/m^2^) on day 1 and fluorouracil (2400 mg/m^2^) on days 1–2 of a 21-day cycle. The DCF regimen consisted of docetaxel (70 mg/m^2^) on day 1, cisplatin (70 mg/m^2^) on day 1, and fluorouracil (750 mg/m^2^) on days 1–5 of a 21-day cycle. Patients were scheduled to undergo two courses of FP, four courses of FOLFOX, or three courses of DCF, decided upon by oncologists according to the patients’ physical condition and disease stage. Esophagectomy with three-field lymph node dissection was performed via open or minimally invasive surgery. The extent of lymph node dissection was similar for open thoracotomy and minimally invasive thoracoscopy. Esophageal reconstruction was usually performed via the substernal route using a gastric tube. In cases with a history of laparotomy, open surgery was performed.

### Skeletal muscle mass loss during NAC

The skeletal muscle mass index (SMI^14^) was used as a skeletal muscle mass indicator, which was calculated from computed tomography (CT) images at the level of L3. CT was performed twice in clinical practice, within 1 month before NAC and within 2 weeks after the last cycle of NAC. The cross-sectional area (−29 to 150 Hounsfield units) on CT images was measured in the skeletal muscle area using SliceOmatic (Image Labo, Saitama, Japan).^14^ The SMI was calculated as cross-sectional skeletal muscle area ÷ (height^2^). The SMI loss (ΔSMI) was used a primary endpoint, which was calculated as pre-NAC minus post-NAC SMI.

### Progression of physical frailty during NAC

We measured a physical function indicator and a disability indicator to evaluate physical frailty. The incremental shuttle walking test (ISWT)^15^ and World Health Organization Disability Assessment Schedule (WHODAS) version 2.0,^16^ within 4 weeks before starting NAC and within 3 weeks after last cycle of NAC, were measured as physical function and disability. The decline in ISWT (ΔISWT = pre-NAC value − post-NAC value) and worsening of WHODAS (ΔWHODAS = post-NAC value − pre-NAC value) were calculated as progression of physical frailty.

### Baseline factors

We also obtained the following information before NAC: age, sex, body mass index (BMI), high Charlson comorbidity index (CCI, ≥1),^4^ low-G8 (≤14) score,^17^ high Cancer and Aging Research Group (CARG, ≥10) score,^18^ clinical stage (≥III),^4^ clinical T factor (≥3)^4^ and clinical N factor (positive)^4^ according to UICC–TNM classification 8th edition, high C-reactive protein (CRP, ≥0.5 mg/dL),^4^ high neutrophil-to-lymphocyte ratio (NLR, ≥3.5),^4^ percent vital capacity (%VC), percent forced expiratory volume in 1 second (%FEV), moderate to vigorous physical activity (MVPA) and sitting time evaluated using Global Physical Activity Questionnaire (GPAQ),^19^ and Geriatric Nutritional Risk Index (GNRI).^20^ All factors were measured within 4 weeks before starting NAC. Cachexia at pretreatment was defined as BMI <21 kg/m^2^ and presence of subdomain: CRP >0.5 mg/dL or presence of anorexia (National Cancer Institute Patient-Reported Outcome Common Terminology Criteria for Adverse Events version 1 [PRO-CTCAE]),^21^ considering the Asian Working Group for Cachexia criteria.^22^

### During-NAC and post-NAC factors

We obtained the following information after NAC: low average relative dose intensity (RDI, <85),^23^ high histological response of NAC (≥1b),^24^ change in ΔCRP, ΔNLR, Δ%VC, Δ%FEV, Δsitting time, ΔMVPA, ΔGNRI during NAC (Δ = post-NAC value − pre-NAC value), and presence of hematologic toxicity (grade ≥ 1) during NAC (NCI CTCAE version 5.0).^25^ Aggravation of physical symptoms during NAC was evaluated with PRO-CTCAE,^21^ defined as attaining a higher grade at post-NAC than at pre-NAC in each subdomain. All post-NAC values were measured within 3 weeks after last cycle of NAC. Pathological stage (≥III),^4^ pathological T factor (≥3),^4^ and pathological N factor (positive) according to UICC–TNM classification 8th edition, and postoperative complications including pneumonia, anastomosis leakage, recurrent nerve paralysis, and others (Clavien–Dindo classification grade [CD grade])^26^ within 30 days after surgery were assessed.

### Statistics

Descriptive statistics are presented as the number of people (%) and mean (standard deviation). The difference in SMI between pre-NAC and post-NAC was analyzed with a paired *t* test. We investigated the associated pre-NAC and during-NAC factors with ΔSMI, the primary endpoint in the present study, using a multivariate regression model to hypothesize the mechanism of skeletal muscle mass loss during NAC. The sample size was interpreted as 80 included patients, considering 10% excursion rate and potential use of seven explanatory variables (biological baseline factors, tumor factor, inflammatory factor, cancer treatment factor, toxicity factor, physical activity factor, and malnutrition-related factor) in multivariate regression analysis, given that this is an exploratory cohort study. Biological baseline factors (age, sex, G8, CARG score), tumor factors (clinical stage, T and N factors), inflammatory factors (CRP, NLR, ΔCRP, ΔNLR), cancer treatment factors (regimen, RDI, histological response), toxicity factors (hepatotoxicity, physical symptoms), physical activity factors (MVPA, sitting time, ΔMVPA, Δsitting time), and nutrition factors (GNRI, ΔGNRI) were used as potential factors for loss of skeletal muscle mass^27–30^ in univariate analysis. Significant factors associated with ΔSMI were detected with a multivariate regression model, using potential factors with *P* <0.1 in univariate analysis and confounding factors (age, sex, regimen of NAC, and pre-NAC SMI). The characteristics of the significant associated factors were analyzed using a one-way analysis of variance or χ^2^ test. If the significant factor was a continuous variable, patients were then divided into two groups using the median value. To confirm that skeletal muscle mass loss during chemotherapy in older patients is a true physical frailty indicator, the associations of ΔSMI with ΔISWT and ΔWHODAS were analyzed using a multivariate regression model adjusted for age, sex, pre-NAC SMI, G8, clinical stage, regimen of NAC, RDI, histological response, and change in MVPA and GNRI during NAC. Statistical significance was considered as two-tailed *P* <0.05. All analyses were performed with R version 4.0.2 (R Foundation for Statistical Computing, Vienna, Austria).

## RESULTS

### Significant factors associated with skeletal muscle mass loss

**Fig. 1:**
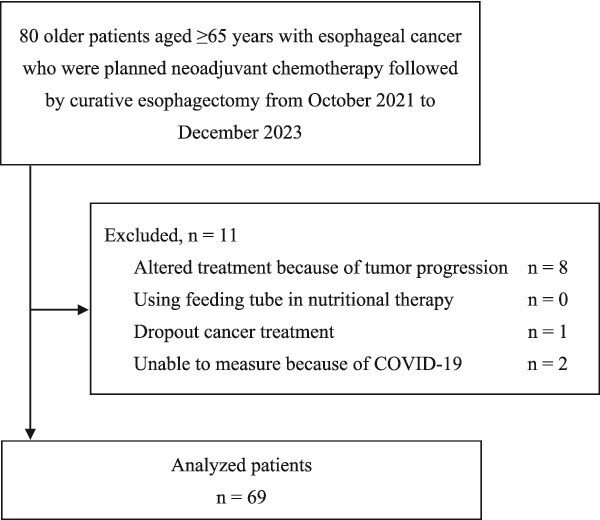
Recruitment flow diagram.

**Fig. 2:**
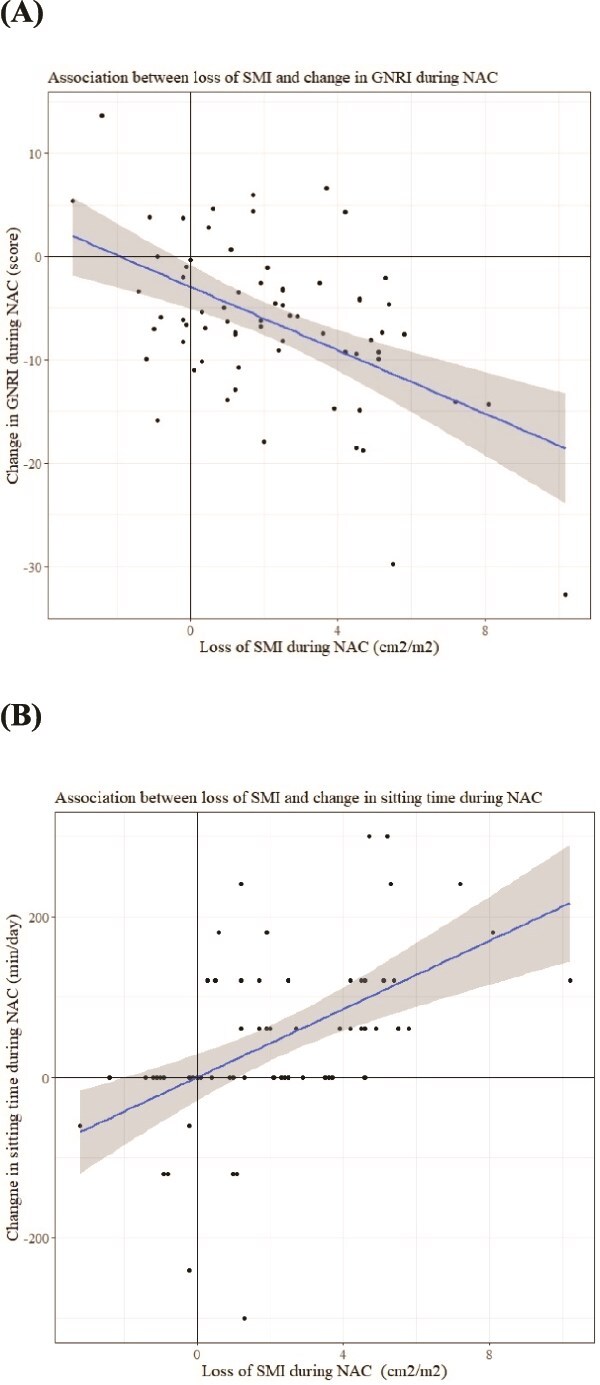
Association of loss of SMI with increase in sitting time and decrease in GNRI during NAC. Scatterplots and regression lines (gray areas: 95% confidence interval) of loss of SMI and increase in sitting time during NAC is indicated in (A), and loss of SMI and decrease in GNRI during NAC is indicated in (B).

A total of 69 patients were analyzed (Fig. 1). The mean SMI before and after NAC was 43.1 (SD 7.8) and 40.9 (7.6) cm^2^/m^2^, respectively; there was a significant difference (*P* < 0.001). The mean ΔSMI was 2.2 (2.6) cm^2^/m^2^. The mean ISWT before and after NAC was 418.7 (136.7) and 353.5 (133.8) m, and the mean ΔISWT was 65.2 (71.4) m. The mean WHODAS score before and after NAC was 13.6 (3.8) and 16.2 (6.9), and the mean ΔWHODAS was 2.7 (2.0). The mean age was 72.9 years, and DCF, FP, and FOLFOX regimens were administered to 46 (67%), 4 (6%), and 19 (27%) patients, respectively (Table 1). The mean BMI was 21.9 (2.7) kg/m^2^ before NAC and 21.3 (2.8) kg/m^2^ after NAC. The numbers of patients with response grade 0, 1a, and 1b-3 were 4 (6%), 20 (29%), and 45 (65%), respectively. In multivariate analysis, significant factors associated with loss of SMI were Δsitting time (per 1 min/day, adjusted coefficient 0.007, 95% confidence interval 0.001 to 0.013, *P* = 0.016), ΔGNRI (per 1 score, adjusted coefficient: −0.146, 95% confidence interval −0.213 to −0.013, *P* = 0.002), and worsening decreased appetite (vs. no worsening, adjusted coefficient 1.571, 95% confidence interval 0.279 to 2.862, *P* = 0.018) (Table 1 and Fig. 2).

**Table 1:** Association of all factors with skeletal muscle mass loss during NAC in univariate analysis

Variables		Values	Univariate analysis		Multivariate analysis			
			β (95% CI)	*P*	β (95% CI)	|Β|	VIF	*P*
Pre-NAC factors	Age (years)	72.9 ± 4.4	−0.092 (−0.232 to 0.049)	0.197	0.028 (−0.102 to 0.159)	0.049	1.576	0.665
	Male	53 (77%)	−0.242 (−1.227 to 1.711)	0.743	−1.200 (−2.609 to 0.208)	0.199	1.714	0.093
	DCF regimen	46 (67%)	0.967 (−0.328 to 2.263)	0.141	−0.429 (−1.653 to 0.794)	0.079	1.613	0.485
	SMI (cm^2^/m^2^)	43.1 ± 7.8	0.083 (0.005 to 0.160)	0.037^*^	0.054 (−0.019 to 0.128)	0.165	1.565	0.144
	Low-G8 (≤14)	41 (67%)	−0.839 (−2.140 to 0.461)	0.200				
	High CARG (≥10)	13 (21%)	−0.207 (−1.647 to 1.232)	0.770				
	High CCI (≥1)	25 (36%)	0.728 (−0.551 to 2.007)	0.260				
	cStage ≥III	56 (81%)	0.620 (−0.960 to 2.200)	0.437				
	cT factor ≥ 3	50 (72%)	0.125 (−1.264 to 1.513)	0.859				
	cN factor positive	59 (86%)	0.772 (−0.981 to 2.534)	0.383				
	High-CRP (≥0.5 mg/dL)	19 (28%)	−0.350 (−1.736 to 1.037)	0.620				
	High-NLR (≥3.5)	36 (52%)	−0.271 (−1.512 to 0.969)	0.660				
	Cachexia	11 (16%)	−1.003 (−2.681 to 0.674)	0.237				
	MVPA (min/week)	384.6 ± 397.2	−0.001 (−0.002 to 0.001)	0.470				
	Sitting time (min/day)	454.4 ± 212.6	0.001 (−0.002 to 0.004)	0.501				
	GNRI (score)	101.0 ± 9.5	0.029 (−0.037 to 0.094)	0.380				
During-NAC factors	Low-RDI (<85%)	44 (64%)	−0.345 (−1.634 to 0.943)	0.590				
	High-response grade (≥1b)	45 (65%)	0.748 (−0.542 to 2.038)	0.251				
	ΔCRP (mg/dL)	0.40 ± 2.52	0.188 (−0.060 to 0.431)	0.130				
	ΔNLR (ratio)	−1.79 ± 2.60	−0.130 (−0.109 to 0.368)	0.280				
	ΔMVPA (min/week)	−106 ± 348	−0.001 (−0.003 to 0.001)	0.200				
	Δsitting time (min/day)	40 ± 105	0.012 (0.007 to 0.175)	<0.001^**^	0.007 (0.001 to 0.013)	0.288	1.303	0.016^*^
	ΔGNRI (score)	−6.3 ± 7.7	−0.169 (−0.238 to −0.099)	<0.001^**^	−0.146 (−0.213 to −0.013)	0.440	1.710	<0.001^**^
	Presence of hematotoxicity							
	Decreasing Hb	23 (33%)	0.083 (−1.234 to 1.400)	0.900				
	Decreasing WBC	52 (75%)	0.871 (−0.554 to 2.295)	0.227				
	Decreasing plate	8 (12%)	0.928 (−0.997 to 2.8529)	0.340				
	Neutropenia	47 (68%)	0.818 (−0.499 to 2.134)	0.219				
	Febrile neutropenia	8 (12%)	−0.811 (−2.739 to 1.117)	0.400				
	Presence of worsening symptom							
	Difficulty swallowing	12 (17%)	1.669 (0.083 to 3.255)	0.039^*^	0.727 (−0.727 to 2.180)	0.108	1.473	0.321
	Mouth/throat sores	23 (33%)	1.491 (0.226 to 2.756)	0.022^*^	−0.432 (−1.559 to 0.695)	0.080	1.368	0.446
	Taste changes	35 (51%)	1.978 (0.834 to 3.121)	<0.001^**^	0.898 (−0.272 to 2.068)	0.176	1.659	0.130
	Decreased appetite	23 (33%)	2.450 (1.277 to 3.623)	<0.001^**^	1.571 (−0.279 to 2.862)	0.291	1.796	0.018^*^
	Nausea	12 (17%)	0.890 (−0.730 to 2.515)	0.280				
	Vomiting	4 (6%)	−0.217 (−2.872 to 2.438)	0.870				
	Constipation	22 (32%)	0.684 (−0.637 to 2.005)	0.310				
	Diarrhea	10 (14%)	0.515 (−1.243 to 2.273)	0.560				
	Shortness of breath	20 (29%)	2.204 (0.946 to 3.462)	<0.001^**^	0.367 (−1.140 to 1.874)	0.065	2.266	0.627
	Fatigue	29 (42%)	1.629 (0.486 to 2.822)	0.008^**^	−0.969 (−2.346 to 0.408)	0.188	2.240	0.164

^*^ *P* < 0.05;

^**^ *P* < 0.01.

Abbreviations: β, regression coefficient; CARG, Cancer and Aging Research Group; CI, confidence interval; CCI, Charlson comorbidity index; CRP, C-reactive protein; DCF, docetaxel + cisplatin + fluorouracil; %FEV, % force expiratory volume in 1 second; GNRI, geriatric nutritional risk index; Hb, hemoglobin; MVPA, moderate to vigorous physical activity; NAC, neoadjuvant chemotherapy; NLR, neutrophil-to-lymphocyte ratio; RDI, relative dose intensity; SMI, skeletal muscle mass index; %VC, % vital capacity; WBC, white blood cell.

### Characteristics of significant factors associated with loss of skeletal muscle mass

There were significant differences in age, regimen, pre-NAC WHODAS, ΔMVPA, Δsitting time, ΔGNRI, ΔISWT, ΔWHODAS, ΔSMI, hematotoxicity of white blood cells (WBCs), presence of worsening mouth/throat sores, taste changes, shortness of breath, fatigue, number of worsening symptoms, and postoperative pneumonia between the groups of patients with non-large increase in sitting time (increase < median 60 min/week, *n* = 34) and large increase in sitting time (increase ≥ median 60 min/week, *n* = 35) (Table 2). There were significant differences in age, regimen, %VC, GNRI, ISWT, pre-NAC WHODAS, ΔCRP, Δsitting time, ΔGNRI, ΔISWT, ΔSMI, and hematotoxicity of WBCs between the groups of patients with non-large decrease in GNRI (score decrease <6.7, *n* = 31) and large decrease in GNRI (score decrease ≥6.7, *n* = 30) (Table 3). There were significant differences in Δsitting time, ΔISWT, ΔWHODAS, ΔSMI, presence of worsening difficulty in swallowing, mouth/throat sores, taste changes, decreased appetite, nausea, shortness of breath, fatigue, number of worsening symptoms, and postoperative pneumonia between the groups of patients with worsening appetite (*n* = 46) and no worsening appetite (*n* = 23) (Table 4).

**Table 2:** Characteristics of the patients with large increase in sitting time during NAC (increase ≥60 min/day)

Variables		Large increase in sitting time	Non-large increase in sitting time	
		*n* = 35	*n* = 34	*P*
Pre-NAC factors	Age (years)	71.7 ± 4.1	74.2 ± 4.34	0.016^**^
	Male	24 (69%)	29 (85.3)	0.174
	DCF regimen	29 (83%)	17 (50.0)	0.008^*^
	SMI (cm^2^/m^2^)	44.0 ± 7.7	42.1 ± 7.9	0.303
	Low-G8 (≤14)	22 (63%)	24 (71%)	0.670
	High CARG (≥10)	8 (23%)	9 (26%)	0.945
	High CCI (≥1)	13 (37%)	12 (35%)	1.000
	cStage ≥ III	30 (86%)	26 (76%)	0.500
	cT factor ≥ 3	27 (77%)	23 (68%)	0.540
	cN factor positive	32 (91%)	27 (80%)	0.282
	Cachexia	4 (11%)	7 (21%)	0.478
	High-CRP (≥0.5 mg/dL)	12 (34%)	7 (21%)	0.315
	High-NLR (≥3.5)	17 (49%)	19 (56%)	0.714
	%VC (%)	107.1 ± 16.5	107.3 ± 18.9	0.951
	%FEV (%)	78.8 ± 9.2	75.6 ± 14.6	0.287
	MVPA (min/week)	341 ± 349	429 ± 442	0.366
	Sitting time (min/week)	457 ± 230	468 ± 196	0.835
	GNRI (score)	100.4 ± 8.9	101.5 ± 10.2	0.651
	ISWT (m)	433 ± 150	403 ± 122	0.368
	WHODAS (score)	14.7 ± 4.7	12.4 ± 2.1	0.015^*^
During-NAC factors	Low-RDI (<85%)	21 (60%)	23 (68%)	0.682
	High-response grade (≥1b)	22 (63%)	23 (68%)	0.869
	ΔCRP (mg/dL)	0.47 ± 2.84	0.33 ± 2.20	0.818
	ΔNLR (ratio)	−1.36 ± 1.74	−2.24 ± 3.23	0.164
	Δ%VC (%)	−2.8 ± 10.9	2.9 ± 20.7	0.159
	Δ%FEV (%)	−2.9 ± 10.6	−3.1 ± 10.4	0.942
	ΔMVPA (min/week)	−188 ± 343	−21 ± 338	0.046^*^
	Δsitting time (min/day)	123 ± 70	−33 ± 73	<0.001^**^
	ΔGNRI (score)	−8.6 ± 8.6	−3.9 ± 6.0	0.010^*^
	Decline in ISWT (m)	105 ± 69	24 ± 47	<0.001^**^
	Worsening of WHODAS (score)	4.4 ± 5.9	0.8 ± 2.1	0.002^**^
	Loss of SMI (cm^2^/m^2^)	3.6 ± 2.3	0.7 ± 1.8	<0.001^**^
	Presence of hematotoxicity			
	Decreasing Hb	16 (46%)	7 (21%)	0.050
	Decreasing WBC	31 (89%)	21 (62%)	0.021^*^
	Decreasing plate	5 (14%)	3 (9%)	0.740
	Neutropenia	28 (80%)	19 (56%)	0.059
	Febrile neutropenia	5 (14%)	3 (9%)	0.740
	Presence of worsening symptom			
	Difficulty swallowing	8 (23%)	4 (12%)	0.369
	Mouth/throat sores	18 (51%)	5 (15%)	0.003^**^
	Taste changes	24 (69%)	11 (32%)	0.006^**^
	Decreased appetite	15 (43%)	8 (23%)	0.148
	Nausea	8 (23%)	4 (11%)	0.369
	Vomiting	3 (9%)	1 (3%)	0.627
	Constipation	12 (34%)	10 (29%)	0.860
	Diarrhea	7 (20%)	3 (9%)	0.329
	Shortness of breath	18 (51%)	2 (6%)	<0.001^**^
	Fatigue	21 (60%)	8 (23%)	0.005^**^
	Number of worsening symptoms	4.5 ± 2.9	2.1 ± 2.3	<0.001^**^
Postoperative factors	pStage ≥ III	15 (43%)	14 (41%)	1.000
	pT factor ≥ 3	13 (38%)	13 (38%)	1.000
	pN factor positive	19 (56%)	15 (44%)	0.467
	Complications CD grade ≥ III	6 (17%)	6 (18%)	1.000
	Anastomosis leakage	4 (11%)	1 (6%)	0.696
	Pneumonia	13 (37%)	4 (12%)	0.030^*^
	Recurrent nerve paralysis	12 (34%)	7 (21%)	0.315

^*^ *P* < 0.05;

^**^ *P* < 0.01.

Abbreviations: CARG, Cancer and Aging Research Group; CI, confidence interval; CCI, Charlson comorbidity index; CD, Clavien–Dindo classification; CRP, C-reactive protein; DCF, docetaxel + cisplatin + fluorouracil; %FEV, % force expiratory volume in 1 second; GNRI, Geriatric Nutritional Risk Index; Hb, hemoglobin; ISWT, incremental shuttle walking test; MVPA, moderate to vigorous physical activity; NAC, neoadjuvant chemotherapy; NLR, neutrophil-to-lymphocyte ratio; RDI, relative dose intensity; SMI, skeletal muscle mass index; %VC, % vital capacity; WBC, white blood cell; WHODAS, WHO Disability Assessment Schedule.

**Table 3:** Characteristics of the patients with large decrease in GNRI during NAC (score decrease ≥6.25)

Variables		Large decrease in GNRI	Non-large decrease in GNRI	
		*n* = 34	*n* = 35	*P*
pre-NAC factors	Age (years)	71.1 ± 3.42	74.7 ± 4.6	0.001^**^
	Male	28 (82.4%)	25 (71%)	0.430
	DCF regimen	29 (85%)	17 (49%)	0.003^**^
	SMI (cm^2^/m^2^)	44.3 ± 8.6	41.9 ± 6.8	0.197
	Low-G8 (≤14)	19 (56%)	27 (77%)	0.106
	High CARG (≥10)	6 (18%)	11 (31%)	0.294
	High CCI (≥1)	11 (32%)	14 (40%)	0.682
	cStage ≥ III	28 (82%)	28 (80%)	1.000
	cT factor ≥ 3	23 (68%)	27 (77%)	0.540
	cN factor positive	30 (88%)	29 (83%)	0.770
	Cachexia	5 (15%)	6 (17%)	1.000
	High-CRP (≥0.5 mg/dL)	7 (21%)	12 (34%)	0.315
	High-NLR (≥3.5)	15 (44%)	21 (60%)	0.280
	%VC (%)	111.7 ± 18.6	102.9 ± 15.5	0.037^*^
	%FEV (%)	78.3 ± 10.0	76.1 ± 14.0	0.449
	MVPA (min/week)	357 ± 330	411 ± 456	0.570
	Sitting time (min/day)	473 ± 236	451 ± 189	0.657
	GNRI (score)	103.4 ± 8.4	98.5 ± 10.0	0.030^*^
	ISWT (m)	456 ± 150	382 ± 113	0.024^*^
	WHODAS (score)	14.1 ± 4.7	13.0 ± 2.6	0.238
During-NAC factors	Low-RDI (<85%)	22 (65%)	22 (63%)	1.000
	High-response grade (≥1b)	25 (73%)	20 (57%)	0.240
	ΔCRP (mg/dL)	1.23 ± 2.51	−0.41 ± 2.30	0.006^**^
	ΔNLR (ratio)	−1.45 ± 1.59	−2.1 ± 3.29	0.287
	Δ%VC (%)	0.8 ± 20.8	−0.8 ± 11.5	0.693
	Δ%FEV (%)	−4.50 ± 10.5	−1.56 ± 10.3	0.245
	ΔMVPA (min/week)	−138 ± 369	−74.6 ± 329	0.454
	Δsitting time (min/week)	76 ± 93	17 ± 111	0.020^*^
	ΔGNRI (score)	−11.8 ± 6.1	−0.9 ± 4.7	<0.001^**^
	Decline in ISWT (m)	83 ± 83	48 ± 54	0.045^*^
	Worsening of WHODAS (score)	3.6 ± 6.1	1.8 ± 2.9	0.123
	Loss of SMI (cm^2^/m^2^)	2.9 ± 2.8	1.4 ± 2.1	0.013^*^
	Presence of hematotoxicity			
	Decreasing Hb	11 (32%)	12 (34%)	1.000
	Decreasing WBC	30 (88%)	22 (63%)	0.030^*^
	Decreasing plate	4 (12%)	4 (11%)	1.000
	Neutropenia	27 (79%)	20 (57%)	0.084
	Febrile neutropenia	4 (12%)	4 (11%)	1.000
	Presence of worsening symptom			
	Difficulty swallowing	7 (21%)	5 (14%)	0.709
	Mouth/throat sores	14 (41%)	9 (26%)	0.268
	Taste changes	19 (56%)	16 (46%)	0.546
	Decreased appetite	11 (32%)	12 (34%)	1.000
	Nausea	6 (18%)	6 (17%)	1.000
	Vomiting	1 (3%)	3 (9%)	0.627
	Constipation	10 (29%)	12 (34%)	0.860
	Diarrhea	5 (15%)	5 (14%)	1.000
	Shortness of breath	14 (41%)	6 (17%)	0.053
	Fatigue	15 (44%)	14 (40%)	0.918
	Number of worsening symptoms	3.6 ± 3.2	3.0 ± 2.5	0.376
Postoperative factors	pStage ≥ III	16 (47%)	13 (38%)	0.555
	pT factor ≥ 3	13 (38%)	13 (38%)	1.000
	pN factor positive	17 (50%)	17 (50%)	1.000
	Complications CD grade ≥ III	7 (21%)	5 (14%)	0.709
	Anastomosis leakage	2 (6%)	4 (11%)	0.696
	Pneumonia	11 (32%)	6 (17%)	0.235
	Recurrent nerve paralysis	11 (32%)	8 (23%)	0.540

^*^ *P* < 0.05;

^**^ *P* < 0.01.

Abbreviations: CARG, Cancer and Aging Research Group; CI, confidence interval; CCI, Charlson comorbidity index; CD, Clavien–Dindo classification; CRP, C-reactive protein; DCF, docetaxel + cisplatin + fluorouracil; %FEV, % force expiratory volume in 1 second; GNRI, Geriatric Nutritional Risk Index; Hb, hemoglobin; ISWT, incremental shuttle walking test; MVPA, moderate to vigorous physical activity; NAC, neoadjuvant chemotherapy; NLR, neutrophil-to-lymphocyte ratio; RDI, relative dose intensity; SMI, skeletal muscle mass index; %VC, % vital capacity; WBC, white blood cell; WHODAS, WHO Disability Assessment Schedule.

**Table 4:** Characteristics of the patients with worsening appetite during NAC

Variables		Worsening appetite	Non-worsening appetite	
		*n* = 46	*n* = 23	*P*
pre-NAC factors	Age (years)	73.2 ± 4.5	72.8 ± 4.4	0.745
	Male	20 (87%)	33 (72%)	0.267
	DCF regimen	16 (70%)	30 (65%)	0.928
	SMI (cm^2^/m^2^)	44.2 ± 7.4	42.5 ± 8.0	0.401
	Low-G8 (≤14)	14 (61%)	32 (70%)	0.652
	High CARG (≥10)	6 (26%)	11 (24%)	1.000
	High CCI (≥1)	12 (52%)	13 (28%)	0.092
	cStage ≥ III	17 (74%)	39 (85%)	0.446
	cT factor ≥ 3	15 (65%)	35 (76%)	0.505
	cN factor positive	18 (78%)	41 (89%)	0.397
	Cachexia	10 (21%)	1 (4%)	0.131
	High-CRP (≥0.5 mg/dL)	7 (30%)	12 (26%)	0.924
	High-NLR (≥3.5)	12 (52%)	24 (52%)	1.000
	%VC (%)	106.1 ± 16.6	107.7 ± 18.2	0.720
	%FEV (%)	76.6 ± 13.8	77.5 ± 11.4	0.766
	MVPA (min/week)	391 ± 435	381 ± 382	0.927
	Sitting time (min/day)	469 ± 182	458 ± 228	0.840
	GNRI (score)	103.4 ± 8.3	99.7 ± 9.9	0.132
	ISWT (m)	437 ± 152	409 ± 129	0.437
	WHODAS (score)	14.0 ± 3.0	13.3 ± 4.2	0.507
During-NAC factors	Low-RDI (<85%)	12 (52%)	32 (70%)	0.250
	High-response grade (≥1b)	17 (74%)	28 (61%)	0.421
	ΔCRP (mg/dL)	0.10 ± 1.39	0.55 ± 2.93	0.496
	ΔNLR (ratio)	−1.74 ± 1.81	−1.82 ± 2.94	0.906
	Δ%VC (%)	−3.8 ± 10.4	1.9 ± 18.8	0.183
	Δ%FEV (%)	−4.4 ± 10.4	−2.3 ± 10.5	0.437
	ΔMVPA (min/week)	−168 ± 275	−75 ± 378	0.299
	Δsitting time (min/week)	99 ± 100	19 ± 100	0.003^**^
	ΔGNRI (score)	−7.5 ± 8.1	−5.7 ± 7.5	0.349
	Decline in ISWT (m)	106 ± 76	44 ± 60	<0.001^**^
	Worsening of WHODAS (score)	5.3 ± 6.9	1.3 ± 2.4	0.001^**^
	Loss of SMI (cm^2^/m^2^)	3.8 ± 2.4	1.4 ± 2.2	<0.001^**^
	Presence of hematotoxicity			
	Decreasing Hb	6 (26%)	17 (37%)	0.527
	Decreasing WBC	17 (74%)	35 (76%)	1.000
	Decreasing plate	4 (17%)	4 (9%)	0.506
	Neutropenia	16 (70%)	31 (67%)	1.000
	Febrile neutropenia	3 (13%)	5 (11%)	1.000
	Presence of worsening symptom			
	Difficulty swallowing	8 (35%)	4 (9%)	0.018^*^
	Mouth/throat sores	13 (56%)	10 (22%)	0.009^**^
	Taste changes	20 (87%)	15 (33%)	<0.001^**^
	Decreased appetite	23 (100%)	0 (0%)	<0.001^**^
	Nausea	8 (35%)	4 (9%)	0.018^*^
	Vomiting	3 (13%)	1 (2%)	0.202
	Constipation	9 (39%)	13 (28%)	0.523
	Diarrhea	4 (17%)	6 (13%)	0.904
	Shortness of breath	13 (56%)	7 (15%)	0.001^**^
	Fatigue	18 (78%)	11 (24%)	<0.001^**^
	Number of worsening symptoms	5.9 ± 2.3	2.0 ± 2.1	<0.001^**^
Postoperative factors	pStage ≥ III	10 (43%)	19 (41%)	1.000
	pT factor ≥ 3	5 (23%)	21 (46%)	0.120
	pN factor positive	12 (54%)	22 (52%)	0.795
	Complications CD grade ≥ III	2 (9%)	10 (22%)	0.312
	Anastomosis leakage	2 (9%)	4 (8%)	1.000
	Pneumonia	10 (44%)	7 (15%)	0.023
	Recurrent nerve paralysis	7 (30)	12 (26%)	0.924

^*^ *P* < 0.05;

^**^ *P* < 0.01.

Abbreviations: CARG, Cancer and Aging Research Group; CI, confidence interval; CCI, Charlson comorbidity index; CD, Clavien–Dindo classification; CRP, C-reactive protein; DCF, docetaxel + cisplatin + fluorouracil; %FEV, % force expiratory volume in 1 second; GNRI, Geriatric Nutritional Risk Index; Hb, hemoglobin; ISWT, incremental shuttle walking test; MVPA, moderate to vigorous physical activity; NAC, neoadjuvant chemotherapy; NLR, neutrophil-to-lymphocyte ratio; RDI, relative dose intensity; SMI, skeletal muscle mass index; %VC, % vital capacity; WBC, white blood cells; WHODAS, WHO Disability Assessment Schedule.

### Confirmation of the association of skeletal muscle mass loss with physical frailty progression

Loss of SMI was confirmed to be significantly associated with ΔISWT (per 1 cm^2^/m^2^, adjusted coefficient 19.950, 95% confidence interval 14.299 to 25.601, *P* < 0.001) and ΔWHODAS (per 1 cm^2^/m^2^, adjusted coefficient 1.057, 95% confidence interval 0.553 to 1.560, *P* < 0.001), adjusted for age, sex, pre-NAC SMI, G8, clinical stage, regimen of NAC, RDI, histological response, and change in MVPA and GNRI during NAC (Table 5).

**Table 5:** Association of skeletal muscle mass loss with progression of physical frailty during NAC

	Variables	Association with worsening WHODASΔ		Association with decline in ISWTΔ	
		β (95% CI)	*P*	β (95% CI)	*P*
Pre-NAC factors	Age (years)	−0.085 (−0.401 to 0.231)	0.590	−1.816 (−5.364 to 1.731)	0.308
	Male	2.584 (−0.600 to 5.769)	0.109	11.374 (−24.370 to 47.118)	0.525
	SMI before NAC (cm^2^/m^2^)	−0.027 (−0.213 to 0.158)	0.767	−0.179 (−2.259 to 1.901)	0.863
	Low-G8 (≤14)	2.796 (0.126 to 5.466)	0.040^*^	23.525 (−6.440 to 53.491)	0.121
	cStage ≥ III	−2.237 (−5.427 to 0.954)	0.165	−37.865 (−73.673 to −2.057)	0.039^*^
	DCF regimen	−1.545 (−4.267 to 1.176)	0.259	13.321 (−17.223 to 44.866)	0.385
During-NAC factors	Low-RDI (<85%)	−0.198 (−2.466 to 2.069)	0.861	19.204 (−6.247 to 44.654)	0.136
	High-response grade (≥1b)	1.570 (−0.764 to 3.906)	0.182	−18.803 (−45.006 to 7.401)	0.156
	Change in MVPAΔ	0.001 (−0.002 to 0.004)	0.632	−0.014 (−0.048 to 0.020)	0.421
	Change in GNRIΔ	−0.106 (−2.874 to 0.076)	0.247	−1.247 (−3.286 to 0.791)	0.421
	Loss of SMIΔ (cm^2^/m^2^)	1.057 (0.553 to 1.560)	<0.001^**^	19.950 (14.299 to 25.601)	<0.001^**^

^*^ *P* < 0.05;

^**^ *P* < 0.01.

Abbreviations: β, regression coefficient; CI, confidence interval; DCF, docetaxel + cisplatin + fluorouracil; GNRI, geriatric nutritional risk index; MVPA, moderate to vigorous physical activity; NAC, neoadjuvant chemotherapy; SMI, skeletal muscle mass index.

## DISCUSSION

This prospective cohort study is the first to investigate the factors associated with skeletal muscle mass loss during NAC in older patients with LAEC, with the aim of hypothesizing the clinical mechanism of such loss of skeletal muscle mass during NAC. Currently, aging, disease and inflammation, physical activity, and nutrition are well known to lead to changes in skeletal muscle mass of older patients.^27^ In the field of cancer, treatment-related factors such as mitochondrial toxicity and muscle atrophy, anorexia, nausea, and inactivity caused by chemotherapy, and cancer-related factors such as cachexia, are also well known to influence change in skeletal muscle mass.^28–30^ However, there is no research on which factors truly impact skeletal muscle mass loss during NAC in older patients with LAEC from which to conjecture the actual clinical mechanism. We show in this study that loss of skeletal muscle mass during NAC was associated with increased sitting time among physical activity factors, decline in GNRI among nutritional factors, and appetite loss among physical symptom factors during NAC, with a dose-dependent relationship, in older patients with LAEC. Additionally, these inactivity-related mechanisms and malnutrition-related mechanisms including decline in GNRI and appetite loss were shown to perhaps be caused by a complex of intensity of regimen, physical symptoms, and adverse events of NAC.

Mechanical stress to muscle fibers caused by physical activity, which could alter muscle mass volume via an alteration in the protein synthesis and degradation balance, is an important factor in changes in skeletal muscle mass.^31^^,^^32^ In fact, increased sitting time along with decreased physical activity time was shown to be associated with skeletal muscle mass loss in older adults in a systematic review.^33^ Therefore, in older patients with LAEC, decreased mechanical stress to muscle fibers with increased sitting time may be an especially important factor in the loss of skeletal muscle mass during NAC. In the present study, the group with a large increase in sitting time had significantly higher worsening rates of two chemotherapy-related physical symptoms that induce inactivity, namely, fatigue (60% vs. 23%) and shortness of breath (51% vs. 6%), in comparison with counterparts with no large increase in sitting time. The group with large increase in sitting time also had a significantly higher number of worsening symptoms during NAC than in the non-large increase group (mean 4.5 vs. 2.1 symptoms). Thus, the mechanism for increased sitting time may arise from chemotherapy-related physical symptoms and distress from multiple symptoms^34^ that negatively impact skeletal muscle mass loss during NAC in older patients via decreased mechanical stress to muscle fibers.

Malnutrition-related mechanism during NAC was also one of the main mechanisms for skeletal muscle mass loss. The malnutrition-related mechanism may consist of two pathways, catabolism imbalance caused by chemotherapy and decreased food intake via appetite loss. Compared with the group with non-large decrease in GNRI, the patients with a large decrease in GNRI had significantly higher rates of decreasing WBCs (88% vs. 63%) and had larger increasing ΔCRP (mean 1.23 vs. −0.41 mg/dL) during NAC. It was previously reported that the DCF regimen specifically induced leukocytopenia (84% to 97%) with high incidence rates.^35^^,^^36^ Additionally, the group with a large decrease in GNRI had an overwhelmingly higher rate of DCF regimen (85% vs. 49%) than counterparts with a non-large decrease in GNRI. In recent studies, a multiple anticancer drug regimen was reported to cause treatment-related myotoxicity, which directly impacts skeletal muscle by interference in signal transduction and dysregulation of anabolic–catabolic balance at the tissue level.^37^ Thus, decline in GNRI may have reflected over-catabolism, such as protein wasting with infection and inflammation induced by hematotoxicity and treatment-related myotoxicity, from a high-intensity regimen such as the DCF regimen consisting of three anticancer drugs. Decreased food intake via appetite loss is also an important pathway for the malnutrition-related mechanism. Patients with worsening appetite during NAC had significantly higher rates of worsening difficulty in swallowing (35% vs. 9%), mouth/throat sores (56% vs. 22%), taste changes (87% vs. 33%), nausea (35% vs. 9%), shortness of breath (56% vs. 15%), and fatigue (78% vs. 24%) than those without worsening appetite. The appetite loss may also be caused by multiple factors, including swallowing, oral pain, dysgeusia, inactivity, and fatigue during NAC in the present study.

Tumor-related factors, including cachexia, clinical tumor stage, response to chemotherapy, and inflammatory and immune biomarkers, were not significant for loss of skeletal muscle mass during NAC in older patients. There may be two reasons for this. First, the subjects of the present study had non-metastatic resectable cancer. The stage of the tumor is an important factor for cancer cachexia because cancer cachexia is reported to be caused by increased catabolic mediators arising from tumor overexpression and tumor inflammation.^38^ In metastatic unresectable esophageal cancer, the rate of cachexia was reported to be 52.9%.^39^ In the present study, the rate of cachexia at baseline was 16% in patients with locally advanced esophageal cancer, implying that in this form of cancer the impact of cachexia on loss of skeletal muscle mass during NAC may be weak. Second, the rate of high (grade ≥ 1b) and poor (grade 0) response by chemotherapy was 64% and 6%, respectively, which means that tumor progression was relatively well controlled. Third, there was a diversity of physical vulnerability at baseline because of the older age of the subjects (mean age: 72.9 years). In fact, the rates of vulnerability were 67% in G8, 21% in CARG score, and 36% in CCI, meaning that the subjects were at high risk of toxicity by chemotherapy and had low resilience. Therefore, in older patients with locally advanced esophageal cancer, the change in lifestyle factors resulting from the toxicity of chemotherapy might regulate the loss of skeletal muscle mass during NAC rather than cachexia and tumor progression.

**Fig. 3:**
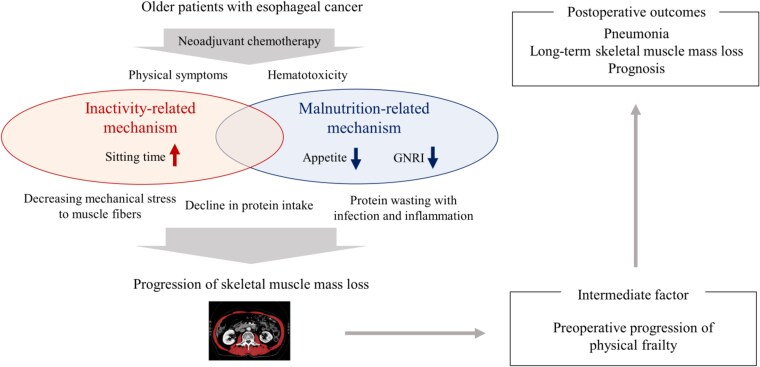
The mechanisms and clinical impacts of the skeletal muscle mass loss during neoadjuvant chemotherapy. The hypothesis that there are two main clinical mechanisms, namely the inactivity-related mechanism and malnutrition-related mechanism, causing loss of skeletal muscle mass and the skeletal muscle mass loss impacts postoperative outcomes through physical frailty progression in older patients with LAEC.

In older patients with cancer, skeletal muscle mass loss during chemotherapy is an important biomarker associated with postoperative complications, health outcomes, and prognosis, which reflected the preoperative physical frailty.^4^^,^^13^^,^^23^ However, there is no global information on whether skeletal muscle mass during NAC truly reflects physical frailty in older patients. In the present study, skeletal muscle mass loss during NAC actually impacted progression of physical frailty during NAC, such as decline in ISWT and worsening of WHODAS score during NAC. To summarize the current study and previous literature, two main clinical mechanisms, namely, the inactivity-related mechanism and malnutrition-related mechanism, cause loss of skeletal muscle mass with physical frailty progression, which may negatively impact postoperative outcomes in older patients with LAEC^4^^,^^13^^,^^22^ (Fig. 3). Hence, if appropriate prehabilitation during NAC to improve lifestyle factors, such as physical activity and nutrition status, is provided to these patients, not only skeletal muscle mass loss during NAC with progression of physical frailty but also poor postoperative outcomes may be preventable. In particular, our group with large increase in sitting time had not only significantly larger decline in ΔISWT (mean 105 vs. 24 m) and larger worse ΔWHODAS (mean 4.4 vs. 0.8 score) but also had a significantly higher rate of postoperative pneumonia (37% vs. 12%) compared with the group with non-large increase in sitting time. Therefore, increased sitting time may be especially important for skeletal muscle mass loss with progression of physical frailty not only during NAC but also for postoperative outcomes, which can also be considered as the critical target for the development of a novel supportive cancer care program during NAC in older patients with LAEC.

There are several limitations in the present study. First, it is a single-center observational study with a small sample size. The causality regarding the association of skeletal muscle mass loss with significant factors during NAC is unclear. The generalizability of these results needs to be confirmed by multicenter studies with larger sample sizes. Second, the present study did not focus on the molecular mechanism of skeletal muscle mass loss during NAC. Recent studies suggested that chemotherapy may cause loss of skeletal muscle mass through the myotoxic molecular mechanism.^29^^,^^37^ Third, there is a lack of information regarding cachexia, malnutrition, and medication, including factors such as muscle strength, body weight loss percentage, polypharmacy, and SGLT2 inhibitors. Cachexia is possibly underestimated because of the lack of information on body weight loss percentage. Future studies need to investigate not only the clinical mechanism but also the molecular mechanism of skeletal muscle mass loss during chemotherapy in older patients with cancer. Finally, our study suggested a dose-dependent association between skeletal muscle mass loss and significant factors, but the optimal cutoff point of the significant factors for clinically defined loss of muscle mass was not clarified. The optimal cutoff point of significant factors will be a requirement for future interventional studies. However, the strong point of the present study is that it revealed significant factors associated with the clinical mechanism of skeletal muscle mass loss during chemotherapy using a prospective study design with informative assessments. As a result, it was suggested that loss of skeletal muscle mass is caused by the inactivity-related mechanism and malnutrition-related mechanism during NAC. Additionally, the inactivity-related mechanism may be especially important for skeletal muscle mass loss with progression of physical frailty during chemotherapy and the risk of postoperative pneumonia. Inactivity and partial physical symptoms including decreased appetite and fatigue can be improved by appropriate exercise therapy, nutritional therapy, and antiemetic drugs. Unfortunately, there is currently a lack of evidence and standard program of prehabilitation during NAC, so establishment of prehabilitation evidence should be a high research priority worldwide.^40^^,^^41^ Altogether, this information will contribute to the development of a novel prehabilitation strategy during NAC and progress in exercise oncology during chemotherapy to improve health and clinical outcomes in geriatric oncology.

In conclusion, this prospective cohort study found an association of increasing sitting time, decreasing GNRI, and appetite loss during NAC with the loss of skeletal muscle mass during NAC in 69 older patients with LAEC. It was hypothesized that the inactivity-related mechanism and malnutrition-related mechanism during NAC are important clinical mechanisms of skeletal muscle mass loss during NAC in older patients with LAEC.

## ABBREVIATIONS

CARG, Cancer and Aging Research Group

CCI, Charlson comorbidity index

CD, Clavien–Dindo classification

CRP, C-reactive protein

CTCAE, Common Terminology Criteria for Adverse Events

DCF, docetaxel + cisplatin + fluorouracil regimen

FEV, forced expiratory volume

FP, cisplatin + fluorouracil regimen

FOLFOX, oxaliplatin + leucovorin + fluorouracil regimen

GNRI, Geriatric Nutritional Risk Index

GPAQ, Global Physical Activity Questionnaire

ISWT, incremental shuttle walking test

LAEC, locally advanced esophageal cancer

MIS, minimally invasive surgery

MVPA, moderate to vigorous physical activity

NAC, neoadjuvant chemotherapy

NLR, neutrophil-to-lymphocyte ratio

RDI, average relative dose intensity

SMI, skeletal muscle mass index

VC, vital capacity

WHODAS, World Health Organization Disability Assessment Schedule

## Notes

Specific author contributions: Data acquisition: Tsuyoshi Harada, Junya Ueno, Nobuko Konishi, Takumi Yanagisawa, Daisuke Kotani, Takashi Kojima, Takeo Fujita; Quality control of data and algorithms: Tsuyoshi Harada, Tetsuya Tsuji, Junya Ueno, Nobuko Konishi, Takumi Yanagisawa, Nanako Hijikata, Aiko Ishikawa, Kakeru Hashimoto, Hitoshi Kagaya, Noriatsu Tatematsu, Sadamoto Zenda; Data analysis and interpretation: Tsuyoshi Harada, Tetsuya Tsuji, Noriatsu Tatematsu, Daisuke Kotani, Takashi Kojima, Takeo Fujita; Statistical analysis: Tsuyoshi Harada; Manuscript preparation: Tsuyoshi Harada, Tetsuya Tsuji; Manuscript review: all authors.
